# 间充质干细胞及其外泌体对肿瘤影响的研究进展

**DOI:** 10.3779/j.issn.1009-3419.2022.101.20

**Published:** 2022-05-20

**Authors:** 舒悦 崔, 帅 汤, 晓玲 丁, 刚 丁

**Affiliations:** 261053 潍坊，潍坊医学院口腔医学院 School of Stomatology, Weifang Medical University, Weifang 261053, China

**Keywords:** 间充质干细胞, 外泌体, 肿瘤, 癌症, Mesenchymal stem cells, Exosome, Tumor, Cancer

## Abstract

在我国，无论城市还是农村，恶性肿瘤都是我国居民死亡的主要原因。间充质干细胞（mesenchymal stem cells, MSCs）具有多向分化潜能、自我更新能力和良好的免疫调节特性。而外泌体作为MSCs旁分泌的重要物质，在肿瘤微环境中介导细胞间的信息交流与传递，影响肿瘤的发生发展。有研究报道了MSCs及其来源的外泌体对肿瘤的影响具有相互矛盾的发现，一方面MSCs及其外泌体具有肿瘤趋向性，能够靶向特定部位抑制肿瘤生长；另一方面也有证据表明MSCs具有致瘤性，作为肿瘤微环境的一部分影响肿瘤的生长和迁移。本文将综述MSCs及其外泌体与肿瘤发生发展的关系以及MSCs及其外泌体如何在肿瘤发生发展中扮演不同角色，以期为肿瘤的诊断、预后和精准治疗提供有益的帮助。

## Abstract (en)

In China, malignant tumor is the main cause of death in both urban and rural areas. Mesenchymal stem cells (MSCs) have multidirectional differentiation potential, self-renewal ability and good immunomodulatory properties. Exosomes, as important paracrine substances of MSCs, mediate information exchange and transmission between cells in tumor microenvironment and influence the occurrence and development of tumors. Recently, conflicting findings have been reported on the effects of MSCs and their exosomes on tumors. On the one hand, MSCs and their exosomes are tumorigenic and can target specific sites to inhibit tumor growth; On the other hand, there is also evidence that MSCs could affect tumor growth and migration as part of the tumor microenvironment. In this paper, we will review the relationship between MSCs and exosomes and tumorgenesis and development, as well as how MSCs and exosomes play different roles in tumorgenesis and development, in order to provide beneficial help for tumor diagnosis, prognosis and precise treatment.

根据世界卫生组织国际癌症研究署发布的2020年全球最新癌症负担数据^[1]^显示：中国癌症新发人数和癌症死亡人数均位居全球第一。研究^[2]^发现，肿瘤微环境对肿瘤的发生发展产生重要影响。间充质干细胞（mesenchymal stem cells, MSCs）由于其良好的多向分化、自我更新潜能以及免疫调节特性，已被广泛应用于多种组织、器官的修复以及再生治疗。研究^[3]^表明，MSCs能够通过旁分泌作用产生外泌体这一生物活性物质，外泌体通过传递多种信号通路影响肿瘤细胞的增殖、迁移和血管生成。目前，在关于MSCs及其外泌体是抑制肿瘤还是促进肿瘤的报道中，出现了不一致的结论。本文将探讨MSCs及其外泌体对肿瘤发生发展的影响及其潜在作用机制。

## 1 间充质干细胞

来源于发育早期中胚层和外胚层的MSCs具有良好的多向分化潜能、自我更新能力和免疫调节性能，已经从骨髓、脂肪、脐带、胎盘、皮肤、外周血、牙齿等多种组织中分离并培养^[4]^。国际细胞治疗学会间充质和组织干细胞委员会提出了定义人类MSCs的最低标准：首先MSCs在标准培养条件下培养时必须贴壁，其次MSCs阳性表达CD105、CD73和CD90，不表达CD45、CD34、CD14或CD11b、CD79a或CD19及HLA-DR表面分子，最后MSCs在体外标准诱导条件下分化为成骨细胞、脂肪细胞和软骨细胞^[5]^。由于MSCs良好的生物学性能，已被应用于多种疾病的基础和临床研究，在心脏病、自身免疫病等疾病中具有良好的临床应用前景^[2]^。MSCs发挥生物学作用主要通过以下机制实现，一是MSCs的迁移和归巢特性，MSCs能够归巢到相应的病变区域；二是旁分泌特性，MSCs通过分泌旁分泌因子促进组织修复与再生；三是免疫调节特性，MSCs通过抑制免疫细胞，如T细胞、B细胞、巨噬细胞等的激活和功能参与免疫调节^[6]^。

## 2 间充质干细胞与肿瘤

MSCs在人体内分布广泛，因此，MSCs在体内会与肿瘤细胞等各种细胞进行接触，能够识别肿瘤的信号并产生相应的反应，同时MSCs会被招募并成为肿瘤微环境的一部分。有研究^[7]^表明，MSCs在肿瘤的发生发展、转移及预后中发挥着重要作用。

### 2.1 肺癌

数据^[1]^显示，肺癌是全球男性和女性癌症死亡的主要原因，同时也是男性最常见的癌症。研究者将人骨髓MSCs与肺癌细胞株A549共培养，结果显示MSCs可以抑制A549细胞的迁移和增殖，并将细胞周期阻滞在G_1_期，同时可以诱导细胞凋亡，MSCs条件培养基取得了相同的效果，然而将MSCs联合A549细胞注入BALB/c小鼠背部皮下，检测肿瘤生长情况，结果显示MSCs组80%-90%形成肉眼可见的肿瘤，且体积明显大于对照组，在MSCs组肿瘤表面分布了丰富的血管，说明在肿瘤微环境中，MSCs促进了血管生成，从而利于肿瘤的生长^[8]^。将MSCs联合Lewis肺癌LL3细胞注入C57BL/6小鼠背部左侧皮下，结果显示联合应用MSCs与LL3细胞可显著促进肿瘤生长。MSCs可抑制腺病毒Fas配体在原发肿瘤中诱导的炎症反应，排除排斥反应，MSCs还可减少肿瘤特异性T细胞的扩增^[9]^。将脐带MSCs与肺癌细胞系H1299共培养后，检测其增殖、凋亡、侵袭及细胞周期，结果显示脐带MSCs能显著抑制H1299细胞侵袭，并诱导H1299细胞凋亡，但对H1299细胞增殖和细胞周期无明显影响，说明脐带MSCs对肺癌细胞具有抗肿瘤的作用，进一步研究^[10]^表明可能是AKT/PI3K/STAT3信号通路在调控过程中起作用。

### 2.2 肝癌

按照全球癌症相关死亡数排序，肝癌已经上升到第二位^[1]^，如不及时诊治，患者的存活概率很低^[11]^。研究者将骨髓MSCs与人肝癌细胞系通过Transwell共培养体系培养，检测肝癌细胞的侵袭和增殖能力，结果^[12]^显示，与MSCs共培养后的肝癌细胞的侵袭能力下降，增殖能力上升。将MSCs和人肝癌细胞H7402细胞注射入SCID小鼠体内，与对照组相比，MSCs治疗组肿瘤形成时间延迟且肿瘤体积明显缩小，肿瘤组织内有广泛的坏死区，可见成纤维细胞样细胞，并在肿瘤区域内可见原位钙化，说明MSCs可以抑制肿瘤生长^[13]^。肝癌裸鼠模型经尾静脉注射MSCs，然后评估血管生成能力，与对照组相比，MSCs治疗组微血管密度增加，促进血管生成，其机制可能与转化生长因子-β1（transforming growth factor-β1, TGF-β1）/Smad信号通路有关^[14]^。

### 2.3 胃癌

胃癌是一种常见的消化道恶性肿瘤，在中国其发病率及病死率均位居恶性肿瘤第三位^[1]^。将人包皮MSCs与胃癌细胞系SGC-7901共培养，体内体外结果显示均抑制胃癌细胞的增殖，并且发现人包皮MSCs条件培养基能够抑制SGC-7901细胞bcl-2的表达，上调bax和caspase-3的表达，提示人包皮MSCs可通过抑制胃癌细胞增殖和促进胃癌细胞凋亡来抑制肿瘤细胞生长^[15]^。将胃癌来源的MSCs与胃癌细胞系BGC-823和MKN-28共培养，结果^[16]^显示：与骨髓MSCs及邻近非癌组织来源MSCs相比，胃癌来源MSCs促进了胃癌细胞的增殖和迁移，促血管生成因子如血管内皮生长因子（vascular endothelial growth factor, VEGF）、巨噬细胞炎性蛋白-2、TGF-β1、白介素（interleukin, IL）-6的表达水平显著升高，进一步研究表明可能是胃癌来源MSCs分泌的IL-8参与了肿瘤的形成，通过中和抗体阻断IL-8的分泌，结果显示削弱了MSCs的成瘤作用，AKT及ERK1/2信号通路参与了此过程。研究^[17]^表明，MSCs能够激活Wnt5a/Ror2信号轴产生CXCL16，进而激活相应的趋化因子受体（chemokine receptor, CXCR）6，通过激活STAT3增加Ror1的表达，最终促进胃癌细胞的增殖和迁移，从而促进肿瘤的进展。

### 2.4 结直肠癌

结直肠癌是严重威胁人类健康的肿瘤之一，在所有肿瘤中其发病率和死亡率分别位于第二位和第五位^[1]^。将过表达CXCR4的MSCs（MSCs-CXCR4）注射到结肠癌小鼠模型，结果显示，注射MSCs-CXCR4小鼠的体重减轻得到缓解，结肠长度延长，肿瘤数目减少，肿瘤负荷减少，结肠组织中的促炎细胞因子水平和STAT3磷酸化水平降低，提示MSC-CXCR4具有有效的抗肿瘤作用^[18]^。另有研究^[19]^表明，注射MSCs可以将结直肠癌细胞周期阻滞于G_1_期从而诱导细胞凋亡，导致体内Wnt和TGF-β-Smad信号通路失调，进而干扰肿瘤的发生。在裸鼠皮下注射结肠癌HCT116靶向肿瘤干细胞，形成异种移植肿瘤，大鼠MSCs治疗后的结果显示，MSCs促进了肿瘤的生长，同时体外实验^[20]^发现大鼠MSCs增加了HCT116靶向肿瘤干细胞的侵袭和迁移。

### 2.5 乳腺癌

2020年，女性乳腺癌首次超过肺癌，成为全球最常见的癌症，在中国女性癌症患者中，发病的主要类型为乳腺癌^[1]^。与乳腺癌细胞MDA-MB-231共培养的MSCs条件培养基，通过抑制STAT3的激活，从而抑制MDA-MB-231细胞的迁移、增殖和血管生成^[21]^。将脐带MSCs与乳腺癌细胞共培养，与单纯乳腺癌细胞组相比，MSCs促进了肿瘤细胞的增殖，且共培养后肿瘤细胞表达间充质细胞的标志物，说明MSCs与肿瘤细胞相互作用促进了肿瘤细胞的生长^[22]^。将小鼠骨髓MSCs与小鼠乳腺癌细胞4T1共培养后可促进4T1细胞增殖，将MSCs与4T1注射到裸鼠体内，结果显示促进肿瘤细胞增殖，肿瘤体积增大，肿瘤的血管面积增多，同时减少了肿瘤中央的坏死^[23]^。

### 2.6 胰腺癌

胰腺癌是消化系统恶性程度最高的肿瘤之一，其临床表现为：隐匿性、进展快、预后差^[24]^。将表达NK4的MSCs与胰腺癌细胞株SW1990共培养后发现，对SW1990细胞的增殖和迁移有明显的抑制^[25]^。肿瘤坏死因子相关凋亡诱导配体工程化的胰腺来源的MSCs能够显著抑制胰腺癌细胞的生长^[26]^。MSCs能够通过上调双调蛋白促进胰腺癌细胞的局部侵袭，特异性小干扰RNA阻断双调蛋白的产生后消除了胰腺癌细胞的局部侵袭，说明双调蛋白可能参与了这个过程^[27]^。来源于MSCs的肌成纤维样细胞能够调节癌细胞内胚层的状态，提高各种干细胞相关基因的表达水平，增强胰腺癌细胞形成球体的能力，促进小鼠胰腺癌肿块的形成，增加对抗癌药物的耐药性，促进胰腺癌的进展^[28]^。

以上研究结果表明，MSCs与肿瘤细胞相互作用可以调节肿瘤微环境，一方面MSCs由于其归巢特性能够有效地进入肿瘤微环境，通过激活多种信号通路抑制肿瘤生长，克服了药物传递效率低及肿瘤微环境渗透的限制，因此，移植预编辑与修饰后的MSCs成为治疗肿瘤的新手段^[29]^；另一方面MSCs通过重塑肿瘤微环境产生支持组织新生血管、肿瘤侵袭和转移的肿瘤细胞生态位，促进肿瘤的进展^[30]^。基于以上因素，更深入地了解MSCs与肿瘤细胞的串扰将成为未来肿瘤治疗的新策略。

## 3 外泌体

细胞外囊泡（extracellular vesicles, EVs）是由多种哺乳动物细胞主动释放的纳米级胞外小泡，根据其生物起源、大小、生物物理特性可分为外泌体、微囊泡及凋亡小体。外泌体自1983年首次在绵羊网织红细胞中发现，最初被认为是细胞碎片或细胞废弃物，它的作用一直被低估，研究^[31]^发现外泌体是细胞间通讯的重要载体。外泌体是一种由脂质双分子层包裹的膜性囊泡，直径30 nm-200 nm，主要参与细胞之间的物质交换和信号转导，外泌体内容物丰富，包括蛋白质、核酸和脂质等在内的生物活性分子，并通过这些生物活性分子调节受体细胞的生物活性，microRNA（miRNA）被认为是外泌体的重要组成部分^[32]^。MSCs来源的外泌体具有与母细胞相似的生物学功能，但与MSCs相比具有以下优势：①外泌体稳定，可长期保存在-80 oC，内容物不易降解；②外泌体更安全，不存在异倍体的可能性；③外泌体可以跨膜转运，直接靶向作用于病变部位；④“无细胞”治疗，降低了免疫排斥的风险，致瘤性降低，解决了MSCs在体内存活率低的问题^[6, 33]^。

## 4 外泌体与肿瘤

有研究^[34]^发现，MSCs发挥作用主要是由于其旁分泌机制，而外泌体作为旁分泌机制的重要因子在肿瘤发生发展及治疗方面受到越来越多的关注。有研究^[35]^表明，外泌体在肿瘤免疫中起着双刃剑的作用，在肿瘤微环境中，外泌体能够在肿瘤细胞、免疫细胞和其他细胞之间传递生物活性分子，帮助癌细胞逃避免疫监视诱导免疫耐受，同时，来自免疫细胞的外泌体能够发挥抑制肿瘤生长、增殖和转移的作用。

### 4.1 肝癌

收集来自肝癌细胞系Hep3B、HuH7来源的癌症干细胞（cancer stem cells, CSCs），用骨髓MSCs来源的外泌体处理CSCs后，降低了Hep3B-CSCs和HuH7-CSCs的增殖、迁移、侵袭、血管生成和自我更新能力。裸鼠皮下注射Hep3B-CSCs诱导成瘤，经骨髓MSCs外泌体治疗后显示，外泌体可抑制CSCs形成的异种移植瘤的生长速度和重量，进一步研究表明外泌体通过C5orf66-AS1/miR-127-3p/DUSP1/ERK轴阻断肝癌细胞的恶性行为^[36]^。将肝癌CSCs和骨髓MSCs来源的外泌体分别注射到二乙基亚硝胺诱导的Albino大鼠肝癌模型，与注射磷酸盐缓冲溶液的对照组相比，肝癌CSCs可显著增加肝脏相对重量和血清肿瘤标志物以及肝酶水平，肝癌标志物GST-P的免疫染色增强，肿瘤结节的数量和面积增加，肿瘤细胞凋亡减少（bax和p53表达下调，bcl2表达上调），血管生成活性增强，转移和侵袭性增强（P13K和ERK蛋白及其下游靶MMP9表达上调，TIMP1 mRNA和蛋白表达下调），并诱导上皮间质转化。但是注射MSCs来源的外泌体后，这些功能的改变都被逆转，说明CSCs和MSCs外泌体在体内分别诱导和抑制肿瘤的发生发展，提示MSCs外泌体在调节肿瘤中发挥着重要作用^[37]^。将N1S1大鼠肝癌细胞接种至肝左叶诱导肿瘤，经脂肪MSCs来源的外泌体治疗后，促进了大鼠自然杀伤细胞的抗肿瘤反应，从而抑制了肝癌发展^[38]^。

### 4.2 肺癌

将骨髓MSCs来源的外泌体与肺癌细胞共培养，结果表明MSCs外泌体miR-190a-5p通过靶向抑制Krüppel样因子15抑制肺癌细胞的迁移和侵袭，从而为治疗肺癌提供新思路^[39]^。将负载外源miR-130a-3p的人脐带MSCs来源的外泌体与肺癌A549细胞共培养，结果显示肺癌细胞能够成功摄取外源miR-130a-3p，并抑制肺癌细胞的增殖、迁移，诱导A549细胞凋亡，提示MSCs外泌体可作为一种miRNA载体在肿瘤治疗中发挥作用^[40]^。将来源于缺氧骨髓MSCs的外泌体与肺癌细胞共培养，结果显示外泌体能够被邻近的癌细胞摄取并促进癌细胞的侵袭和上皮间充质转化，进一步研究^[41]^表明缺氧骨髓MSCs来源的外泌体通过特定的miRNA激活STAT3信号通路诱导上皮间充质转化从而促进肺癌细胞的侵袭。

### 4.3 胃癌

将Lipocalin型前列腺素D2合成酶（Lipocalin type prostaglandin 2 synthase, L-PGDS）转染MSCs，分离富含L-PGDS的EVs。在体外，EVs-L-PGDS与胃癌细胞SGC-7901共培养后可内化并抑制胃癌细胞SGC-7901的克隆形成、迁移和侵袭能力，并促进细胞的凋亡。在体内，裸鼠皮下肿瘤模型注射EVs-L-PGDS后抑制了移植瘤的生长，进一步研究^[42]^表明胃癌的抑制是通过抑制STAT3的磷酸化来实现的。MSCs来源的外泌体与胃癌细胞系SGC-7901联合植入裸鼠皮下诱导成瘤，结果显示外泌体联合治疗组缩短了体内成瘤的时间，促进肿瘤细胞增殖，肿瘤组织中VEGF、CD34、Ⅷ因子血管生成标志物表达明显高于对照组，肿瘤细胞磷酸化ERK1/2、CXCR4、Bcl-2和VEGF的蛋白水平以及α-SMA、CXCR4、VEGF和MDM2 mRNA的表达均高于对照组，提示MSCs来源的外泌体促进了肿瘤细胞的增殖和转移，促进血管生成，使肿瘤生长速度加快，进一步研究^[43]^表明可能是ERK1/2和p38 MAPK通路的激活参与了此过程。将脐带MSCs来源的外泌体与HGC-27胃癌细胞共培养后发现，外泌体可以通过诱导上皮间充质转化增强HGC-27细胞的迁移和侵袭能力，上调胃癌细胞间充质标志物的表达，增强胃癌细胞的体外致瘤性，可诱导胃癌细胞的干细胞化，进一步研究^[44]^表明脐带MSCs来源的外泌体可能是通过激活Akt信号通路诱导胃癌细胞发生上皮间充质转化并赋予其干性。

### 4.4 结直肠癌

miR-16-5-p已被发现与结直肠癌的进展有关，从转染miR-16-5p的骨髓MSCs中分离外泌体并与结直肠癌细胞共培养，检测到过表达miR-16-5p的骨髓MSCs来源的外泌体能够抑制结直肠癌细胞的增殖、迁移和侵袭，同时能够通过下调ITGA2刺激结直肠癌细胞凋亡。体内实验结果证实，过表达miR-16-5p的MSCs来源的外泌体抑制了结直肠癌的肿瘤生长^[45]^。另有研究^[46]^表明，miR-3940-5p可能是结直肠癌中一个重要的MSCs来源的外泌体miRNA，MSCs来源的外泌体携带miR-3940-5p进入结直肠癌细胞和组织，过表达miR-3940-5p抑制了结直肠癌细胞的上皮间充质转化和侵袭，并抑制了肿瘤的转移，整合素α6的下调和TGF-β1信号缺失参与了这一过程。MSCs产生的EVs携带的miR-222能够通过靶向ATF3，抑制AKT1的转录活性，从而促进大肠癌细胞的恶性侵袭和免疫逃逸，而抑制miR-222在体外能够降低大肠癌细胞的恶性侵袭，在体内能够减少肿瘤的形成和免疫逃逸^[47]^。

### 4.5 乳腺癌

研究^[48]^发现，骨髓MSCs来源的外泌体能够抑制乳腺癌细胞的血管生成，进一步研究发现外泌体miR-100能够通过调节mTOR/HIF-1α信号轴下调乳腺癌细胞中VEGF的表达，从而达到抗肿瘤的目的。将骨髓MSCs来源的外泌体与乳腺癌细胞4T1共培养，测定VEGF的表达情况，结果显示外泌体能够被4T1内化，下调VEGF的表达，建立小鼠肿瘤细胞模型，皮下注射乳腺癌细胞4T1，经外泌体治疗后，肿瘤重量减轻，肿瘤组织中VEGF的mRNA表达降低，组织切片结果显示VEGF和CD31的表达相对较弱，体内体外实验均证实外泌体能够通过抑制肿瘤细胞血管生成抑制肿瘤的进展^[49]^。将从MSCs分化的脂肪细胞中分离的外泌体与乳腺癌细胞MCF7共培养，测定其增殖与迁移能力，结果显示外泌体在体外能够主动被MCF7细胞摄取，并促进MCF7细胞的增殖和迁移，保护MCF7免受血清饥饿或化疗药物诱导的凋亡，而外泌体耗尽后则导致更多的肿瘤细胞凋亡。研究者又利用体内小鼠异种移植模型评估外泌体对肿瘤生长的影响，当使用外泌体释放抑制剂耗竭脂肪细胞外泌体时，结果显示与对照组相比，MSCs分化的脂肪细胞的促肿瘤作用减弱，进一步研究^[50]^发现Hippo信号通路与脂肪细胞外泌体促肿瘤作用有关。

### 4.6 胰腺癌

研究^[51]^表明，hsa-miR-143-3p在人MSCs外泌体中高表达。将hsa-miR-143-3p抑制剂转染人MSCs后收集外泌体，并与人胰腺癌细胞系CFPAC-1共培养，结果显示抑制剂组的活细胞数明显高于对照组，凋亡细胞数明显低于对照组；将hsa-miR-143-3p模拟物转染人MSCs后收集外泌体，与人胰腺癌细胞系CFPAC-1注射到裸鼠体内，结果显示模拟物组肿瘤体积小于对照组且部分区域可见细胞坏死，这些结果表明，人MSCs外泌体中的miR-143-3p可促进胰腺癌CFPAC-1细胞的凋亡，抑制细胞的生长、侵袭和迁移，从而起到抑制肿瘤的作用。骨髓MSCs来源的外泌体能够通过miR-126-3p靶向ADAM9抑制胰腺癌细胞的增殖、迁移和侵袭，提高胰腺癌细胞的凋亡率。体内实验^[52]^证实miR-126-3p转染骨髓MSCs治疗组肿瘤体积较小，提示外泌体miR-126-3p通过靶向ADAM9抑制肿瘤的生长和转移。另有研究^[53]^证实，骨髓MSCs来源的外泌体能够通过circ_0030167靶向miR-338-5p/wif1/Wnt 8/β-catenin抑制胰腺癌细胞的侵袭、增殖和迁移，并降低了肿瘤的干性从而为胰腺癌的治疗提供了新视角。

基于以上研究发现，MSCs来源的外泌体在肿瘤的治疗中也表现出双重作用，外泌体作为药物、mRNA、miRNA的递送载体进行肿瘤治疗，其靶向性和生物相容性具有巨大潜力，同时外泌体参与肿瘤进展与抑制的未知分子机制、人工合成技术仍不成熟也成为外泌体靶向肿瘤治疗不可忽视的问题^[54, 55]^。

## 5 总结与展望

近年来，关于MSCs及MSCs来源的外泌体对肿瘤的作用一直是研究热点，一方面MSCs及其外泌体可以通过抑制与细胞增殖、周期、血管生成的基因的表达，抑制肿瘤的生长和进展；另一方面MSCs通过旁分泌作用产生的miRNA和促肿瘤生成分子改变了与肿瘤生长、血管生成及耐药性相关的信号通路，促进肿瘤进展^[56]^。MSCs及其外泌体对肿瘤的双重作用可能是由于多种因素调节的，如细胞来源、培养条件及MSCs与肿瘤细胞间的相互串扰。尽管MSCs及其外泌体在肿瘤治疗中存在争议，但是科学家们利用MSCs移植前预编辑或者修饰靶向治疗肿瘤、利用外泌体与抗肿瘤药物联合治疗作为肿瘤靶向治疗的理想载体，克服了一些障碍^[34, 56]^。今后，在MSCs及其外泌体用于靶向肿瘤治疗的过程中，应关注MSCs及其外泌体的注射时机、给药途径、细胞类型及特性，同时还应确定有关治疗方案的黄金标准，从而为肿瘤的靶向治疗提供新策略^[56-58]^。
